# PTFE as a Multifunctional Binder for High‐Current‐Density Oxygen Evolution

**DOI:** 10.1002/advs.202408544

**Published:** 2024-09-04

**Authors:** Bohan Deng, Xian He, Peng Du, Wei Zhao, Yuanzheng Long, Zhuting Zhang, Hongyi Liu, Kai Huang, Hui Wu

**Affiliations:** ^1^ State Key Lab of New Ceramics and Fine Processing School of Materials Science and Engineering Tsinghua University Beijing 100084 China; ^2^ State Key Laboratory of Information Photonics and Optical Communications & School of Science Beijing University of Posts and Telecommunications Beijing 100876 China; ^3^ Dongfang Electric (Fujian) Innovation Research Institute Co., Ltd Fuzhou Fujian Province 350108 China

**Keywords:** alkaline water electrolysis, binder, high‐current‐density, oxygen evolution reaction, PTFE

## Abstract

Binder plays a crucial role in constructing high‐performance electrodes for water electrolysis. While most research has been focused on advancing electrocatalysts, the application of binders in electrode design has yet to be fully explored. Herein, the in situ incorporation of polytetrafluoroethylene (PTFE) as a multifunctional binder, which increases electrochemical active sites, enhances mass transfer, and strengthens the mechanical and chemical robustness of oxygen evolution reaction (OER) electrodes, is reported. The NiFe‐LDH@PTFE/NF electrode prepared by co‐deposition of PTFE with NiFe‐layered double hydroxide onto nickel foam demonstrates exceptional long‐term stability with a minimal potential decay rate of 0.034 mV h^−1^ at 500 mA cm^−2^ for 1000 h. The alkaline water electrolyzer utilizing NiFe‐LDH@PTFE/NF requires only 1.584 V at 500 mA cm^−2^ and sustains high energy efficiency over 1000 h under industrial operating conditions. This work opens a new path for stabilizing active sites to obtain durable electrodes for OER as well as other electrocatalytic systems.

## Graphical abstract

This work introduces polytetrafluoroethylene (PTFE) as a binder into oxygen evolution reaction (OER) electrode design. The integration of PTFE strengthens the attachment of the electrocatalyst to the substrate and the mechanical strength of the electrode, providing more active sites for OER and enhancing the stability of the electrode. Meanwhile, PTFE modulates the hydrophilicity of the electrode, improving the mass transfer toward OER.

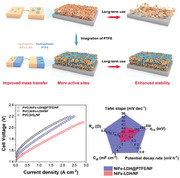

## 1 Introduction

Water splitting to produce green hydrogen is an essential process for generating clean energy, with the promise of reducing dependence on fossil fuels and minimizing carbon emissions.^[^ ^1^ ^]^ However, the energy efficiency of water splitting is hampered by the sluggish oxygen evolution reaction (OER) that occurs at the anode.^[^ ^2^ ^]^ Consequently, improving the performance of the OER is a key research focus in the field of electrochemistry and material science. Decades of research have focused on developing high‐performance OER electrodes through various strategies, such as optimizing the electrocatalysts,^[^ ^3^ ^]^ designing substrates with high surface areas,^[^ ^4^ ^]^ and enhancing the mass transfer of gas products.^[^ ^5^ ^]^ Although numerous OER electrodes with excellent performance were designed under the guidance of these effective strategies, many of them face degradation challenges when applied to industrial applications.^[^ ^6^ ^]^ This is because the operating conditions of industrial water electrolysis place much higher demands on the stability of OER electrodes in many aspects: (i) Mechanical stability. Industrial water electrolysis operates at high current densities (from a few hundred mA cm^−2^ for alkaline [ALK] electrolyzers up to 1–2 A cm^−2^ for anion exchange membrane [AEM] electrolyzers and proton exchange membrane [PEM] electrolyzers),^[^ ^7^ ^]^ during which oxygen bubbles are generated and drastically crack on the surface of OER electrodes. The bonding between the catalyst and the substrate would be disrupted without strong mechanical stability, leading to catalyst detachment.^[^ ^8^ ^]^ (ii) Chemical stability. Industrial water electrolysis operates at high temperatures (60–80 °C) and strong alkaline or acid environments, which can easily cause corrosion if the chemical stability of electrodes is poor.^[^ ^6^, ^9^ ^]^ (iii) Electrochemical stability. As OER electrodes operate at high oxidation potentials, good electrochemical stability is essential for OER electrodes to avoid catalyst agglomeration or dissolution.^[^ ^10^ ^]^ Therefore, designing OER electrodes with excellent mechanical stability, chemical stability, and electrochemical stability is essential for the development of water electrolysis technology.

While most research on OER electrode design has been focused on advancing electrocatalysts, less attention was devoted to the exploration of electrochemical inactive components, such as binders, which could enhance the performance and stability of OER electrodes. Integrating binders into material fabrication is a natural and common strategy when the stability of the material is highly demanded.^[^ ^11^ ^]^ Various types of binders (such as polytetrafluoroethylene [PTFE], PVDF, Nafion, and PANI) have been widely used in various electrochemical applications including Li‐ion battery,^[^ ^12^ ^]^ Metal‐O_2_ battery,^[^ ^13^ ^]^ PEMFC,^[^ ^14^ ^]^ and CO_2_RR.^[^ ^15^ ^]^ However, the incorporation of binders into OER electrode design has been relatively unexplored. While extensive research has been conducted on selecting the most suitable binder types and determining their optimal additive amount,^[^ ^12^, ^14^, ^15^ ^]^ the prevalent method of binder addition typically involves merely mechanically mixing the binder with the catalyst powder, which limits the application of binders. This method is not feasible for self‐supporting electrodes, commonly used in OER and other electrochemical reactions,^[^ ^16^ ^]^ where electrocatalysts are directly grown on the substrate. Furthermore, achieving uniform dispersion of binders can be challenging through mechanical mixing,^[^ ^17^ ^]^ highlighting the need for simultaneous electrocatalyst synthesis and binder incorporation.

**Figure 1:**
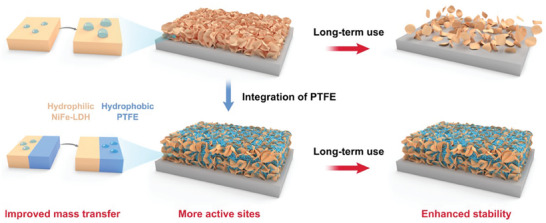
Conceptualization of in situ integrating PTFE as a multifunctional binder into OER electrodes. The integration of PTFE strengthens the attachment of NiFe‐LDH electrocatalyst to the substrate and the mechanical strength of the electrode, providing more active sites for OER and enhancing the stability of the electrode. Meanwhile, it modulates the hydrophilicity of the electrode, improving the mass transfer toward OER.

Herein, we in situ incorporated PTFE as a multifunctional binder with deposited NiFe‐layered double hydroxide (NiFe‐LDH) electrocatalyst toward high‐performance OER (**Figure** **1**). The integration of PTFE enhances the adhesion between the electrocatalyst and substrate and strengthens the mechanical stability of the electrode, stabilizing a greater amount of electrochemical active sites and preventing catalyst detachment during high‐current operations. The high‐temperature and alkali resistance properties of PTFE also augment the chemical and electrochemical stability of the electrode. Meanwhile, PTFE modulates the hydrophilicity of the electrode. The hydrophobic surface provided by PTFE accelerates oxygen desorption from the hydrophilic NiFe‐LDH active sites, thereby improving mass transfer and facilitating the OER process. With the aid of this multifunctional binder, the resultant NiFe‐LDH@PTFE/NF (where “NF” refers to nickel foam) demonstrates superior performance and excellent stability. It achieved low overpotentials of only 228 and 273 mV at 10 and 100 mA cm^−2^, respectively, alongside an impressively low potential decay rate of 0.034 mV h^−1^ at a high current density of 500 mA cm^−2^. Moreover, an alkaline water electrolyzer (AWE) employing Pt/C || NiFe‐LDH@PTFE/NF required only 1.584 V at 500 mA cm^−2^ and sustained high energy efficiency over 1000 hours under industrial operating conditions, demonstrating the potential for industrial application of NiFe‐LDH@PTFE/NF.

## 2 Results and Discussion

### 2.1 Electrode Design and Construction

**Figure 2:**
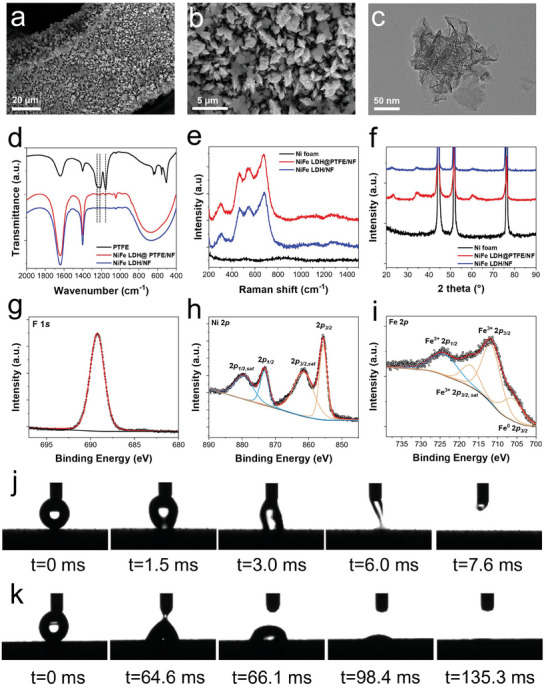
Characterization of the NiFe‐LDH@PTFE/NF electrode. a,b) SEM images and c) TEM image of NiFe‐LDH@PTFE/NF. d) FTIR spectra of PTFE, NiFe‐LDH/NF, and NiFe‐LDH@PTFE/NF. e) XRD pattern and f) Raman spectra of Ni foam, NiFe‐LDH/NF, and NiFe‐LDH@PTFE/NF. g–i) XPS spectra of F 1*s*, Ni 2*p*, and Fe 2*p* regions for NiFe‐LDH@PTFE/NF. j,k) The wetting behavior of water droplets on the surfaces of NiFe‐LDH/NF and NiFe‐LDH@PTFE/NF, illustrating the changes in hydrophilicity due to the integration of PTFE.

PTFE was chosen as the binder for OER electrodes for several reasons. Firstly, PTFE exhibits excellent thermal stability and resistance to acids and alkalis, ensuring its stability under the demanding conditions of industrial water electrolysis. Secondly, as a hydrophobic binder, PTFE enhances mass transfer around the electrocatalyst, which will be discussed in detail later. Thirdly, the low cost of PTFE makes it feasible for large‐scale application in industrial water electrolysis. NiFe‐LDH was selected as a model electrocatalyst to demonstrate the enhancement in performance and stability of OER electrodes provided by PTFE integration, as it is one of the most typical electrocatalysts toward OER.^[^ ^18^ ^]^ The in situ incorporation of PTFE was achieved via a composite electroplating method, which is an effective technique for the incorporation of nano‐sized solid phases in metal coating.^[^ ^19^ ^]^ During this process, nano‐sized solid phases are drawn to the electrode surface through electrostatic interactions, resulting in their concurrent deposition with metal ions onto the electrode surface.^[^ ^20^ ^]^ To prepare the NiFe‐LDH@PTFE/NF electrode, PTFE dispersion was incorporated into the electroplating solution. NiFe‐LDH and PTFE were deposited onto the Ni foam substrate simultaneously through electroplating, resulting in a uniformly coated layer, as shown in the scanning electron microscope (SEM) images (**Figure** **2**a,b). The transmission electron microscope (TEM) image of the NiFe‐LDH@PTFE catalyst is shown in Figure 2c. No agglomerated PTFE particles were found in the SEM and TEM images, while the energy dispersive spectrometer (EDS) analysis confirmed the homogeneous distribution of F element (Figure S1, Supporting Information), indicating a homogenous dispersion of PTFE within the NiFe‐LDH@PTFE/NF. The existence of PTFE in the NiFe‐LDH@PTFE/NF was further confirmed by Fourier transform infrared spectroscopy (FTIR). Compared with the NiFe‐LDH/NF, three new absorption peaks which also appeared in PTFE were found at the NiFe‐LDH@PTFE/NF (Figure 2d). The peaks at 1153 and 1212 cm^−1^ were attributed to asymmetric and symmetric C─F stretching, respectively, while the broad absorption peak at around 1240 cm^−1^ was attributed to an overlap of CF, CF_2_, and CF_3_ vibrations.^[^ ^21^ ^]^

Raman spectroscopy identified the active material without indicating significant changes due to PTFE addition (Figure 2e). The spectra of NiFe‐LDH@PTFE/NF closely matched that of NiFe‐LDH/NF, with peaks at 305, 465, and 548 cm^−1^ associated with the E‐type vibration of Ni─OH lattice, stretching vibration of Ni─O(H), and stretching vibration of Ni─O, respectively.^[^ ^22^ ^]^ Besides, the peak at 678 cm^−1^ was attributed to Fe─O stretching vibration.^[^ ^23^ ^]^ It suggests that the electrochemical process of NiFe‐LDH deposition remains unaffected by PTFE addition. This conclusion was further supported by X‐ray diffraction (XRD) results (Figure 2f). Both NiFe‐LDH@PTFE/NF and NiFe‐LDH/NF showed the same diffraction peaks which were consistent with the hydrotalcite‐like LDH phase (JCPDS no. 38‐0715).

The mass loading of NiFe‐LDH/NF and NiFe‐LDH@PTFE/NF was confirmed by an inductively coupled plasma optical emission spectrometer (ICP‐OES). The Fe content of NiFe‐LDH@PTFE/NF (619 µg cm^−2^) is slightly lower than that of NiFe‐LDH/NF (690 µg cm^−2^), which is consistent with the *I*–*t* profiles during the electroplating process (Figure S2, Supporting Information). Meanwhile, the adsorption surface area of NiFe‐LDH@PTFE/NF (28.91 m^2^ g^−1^) was also slightly lower than that of NiFe‐LDH/NF (31.35 m^2^ g^−1^). The similar pore size distributions between NiFe‐LDH/NF and NiFe‐LDH@PTFE/NF suggest that the integration of PTFE did not significantly change the pore structure of NiFe‐LDH electrocatalyst (Figure S3, Supporting Information).

X‐ray photoelectron spectroscopy (XPS) was performed to characterize the surface chemical composition and chemical states of NiFe‐LDH@PTFE/NF. The F 1*s* spectrum shown in Figure 2g was consistent with the EDS and FTIR results, verifying the existence of PTFE in NiFe‐LDH@PTFE/NF. For Ni 2*p* spectra, fitted peaks at 855.7 and 873.4 eV were attributed to Ni^2+^ 2*p_3/2_* and Ni^2+^ 2*p_1/2_*, and peaks at 861.7 and 879.7 eV were their satellite peaks (Figure 2h). Three fitted peaks shown in Fe 2*p* spectra at 712.4, 724.6, and 717.4 eV were attributed to Fe^3+^ 2*p_3/2_*, Fe^3+^ 2*p_1/2_* and the satellite peak of Fe^3+^ 2*p_3/2_* (Figure 2i). A small peak at 706.7 eV attributed to Fe^0^ 2*p_3/2_* was also found, which is common for NiFe‐LDH.^[^ ^24^ ^]^ Moreover, the chemical states of Ni and Fe in NiFe‐LDH/NF were consistent with that of NiFe‐LDH@PTFE/NF, as shown in Figure S4 (Supporting Information). The hydrophilicity of NiFe‐LDH@PTFE/NF and NiFe‐LDH/NF was evaluated through contact angle measurements. As shown in Figure 2j,k, both electrodes exhibited a superhydrophilic surface due to the hydrophilicity of NiFe‐LDH.^[^ ^25^ ^]^ However, the droplet absorption time for NiFe‐LDH@PTFE/NF was longer than that for NiFe‐LDH/NF, suggesting the influence of PTFE integration.^[^ ^26^ ^]^ All of the above characterizations were in agreement with each other, confirming the successful integration of PTFE into NiFe‐LDH@PTFE/NF without altering the intrinsic properties of NiFe‐LDH, which is good for us to evaluate the effect of PTFE separately.

### 2.2 OER Performance

**Figure 3:**
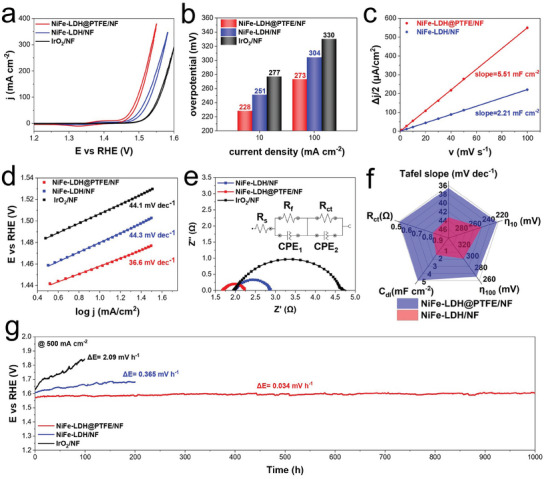
Electrochemical performance of NiFe‐LDH@PTFE/NF electrode in 1 m KOH. a) Cyclic voltammetry (CV) polarization curves of NiFe‐LDH@PTFE/NF, NiFe‐LDH/NF, and a commercial IrO_2_/NF electrode with a scan rate of 1 mV s^−1^. b) Comparison of the overpotentials at 10 and 100 mA cm^−2^ derived from the backward scan in (a). c) Dependence of half current density difference on scan rate, with double‐layer capacitance (*C*_dl_) values estimated by linear fitting. d) Tafel slopes calculated from (a). e) EIS Nyquist plots acquired at 0.6 V versus Hg/HgO with inserted equivalent circuit. f) Comprehensive comparisons between NiFe‐LDH@PTFE/NF and NiFe‐LDH/NF in terms of OER performance. g) Long‐term chronopotentiometry measurement of different electrodes at a constant current density of 500 mA cm^−2^.

The electrocatalytic activity of NiFe‐LDH@PTFE/NF was first evaluated in a three‐electrode system. **Figure** **3**a shows the cyclic voltammetry (CV) profiles of NiFe‐LDH@PTFE/NF, NiFe‐LDH/NF, and a commercial IrO_2_/NF electrode. To exclude the interference of the Ni^2+^/Ni^3+^ oxidation current, current densities during the backward scan were used for the evaluation of electrocatalytic activity. As shown in Figure 3b, NiFe‐LDH@PTFE/NF exhibited lower overpotentials of 228 and 273 mV at 10 and 100 mA cm^−2^, respectively, compared to NiFe‐LDH/NF, which displayed overpotentials of 251 and 301 mV at the same current densities. The superior performance of NiFe‐LDH@PTFE/NF can be attributed to two key factors. First, the integration of PTFE significantly improved the adhesion between the electrocatalyst and substrate, stabilizing more electrocatalysts over the same surface area. This was evidenced by the electrochemical active surface area (ECSA) estimated via the double‐layer capacitance method (Figure 3c and Figure S5, Supporting Information). The *C*_dl_ of NiFe‐LDH@PTFE/NF was 5.51 mF cm^−2^, which was much higher than that of NiFe‐LDH/NF (2.21 mF cm^−2^). It is suggested that more electrochemical active sites were provided with the help of PTFE, resulting in the enhancement of OER activity. Second, the synergistic effect of the hydrophobic PTFE and hydrophilic NiFe‐LDH accelerated oxygen desorption from active sites and water diffusion around the catalyst. Similar structures of a hydrophilic electrocatalyst and a hydrophobic component (like support or additive) have been previously acknowledged for improving mass transfer in OER processes.^[^ ^27^ ^]^ The Tafel slope of NiFe‐LDH@PTFE/NF and NiFe‐LDH/NF were 36.6 and 44.3 mV dec^−1^, respectively (Figure 3d). In situ Raman investigation was conducted to identify the active sites of NiFe‐LDH@PTFE/NF during the OER process. The Raman spectra acquired in the OER region (higher than 0.4 V vs Hg/HgO) displayed a pair of bands at 474 and 553 cm^−1^, which is attributed to Ni─O vibrations in NiOOH (Figure S6, Supporting Information). It is suggested that the active sites in NiFe‐LDH@PTFE/NF are Ni(III)OOH with Fe incorporation, aligning well with the previous study on NiFe‐LDH catalysts.^[^ ^23b^ ^]^ As the intrinsic properties of NiFe‐LDH electrocatalyst remained unchanged according to the in situ Raman investigation and the characterizations discussed above, the faster kinetics of NiFe‐LDH@PTFE/NF was attributed to improved mass transfer facilitated by hydrophobic PTFE. Electrochemical impedance spectroscopy (EIS) was performed to further investigate the OER kinetics. The corresponding parameters of fitted Nyquist plots with respect to the equivalent circuit (inset of Figure 3e) were summarized in Table S1 (Supporting Information). The small semicircle at high frequency represents the film resistance (*R*_f_) while the large semicircle at low frequency represents the charge transfer resistance (*R*_ct_).^[^ ^28^ ^]^ NiFe‐LDH@PTFE/NF showed a lower *R*_ct_ (0.526 Ω) compared to NiFe‐LDH/NF (0.851 Ω), indicating its superior charge and mass transfer capabilities. A comprehensive comparison of OER performance between NiFe‐LDH@PTFE/NF and NiFe‐LDH/NF was summarized in Figure 3f. The comparison between NiFe‐LDH@PTFE/NF and other previously reported OER catalysts was also summarized in Table S2 (Supporting Information). In addition, the influence of PTFE content on OER performance was also investigated. As shown in Figure S7 (Supporting Information), the optimal PTFE concentration in the electroplating solution was around 10–20 g L^−1^. Excessive PTFE content was found to diminish OER activity, especially for high current density regions, underscoring the importance of optimal PTFE levels for maximizing mass transfer during OER.

In addition to enhancing OER activity, the integration of PTFE also significantly improved the stability of NiFe‐LDH@PTFE/NF. As shown in Figure 3g, NiFe‐LDH@PTFE/NF sustained operation at a high current density of 500 mA cm^−2^ for 1000 h, exhibiting a minimal potential decay rate of 0.034 mV h^−1^. In comparison, NiFe‐LDH/NF and the commercial IrO_2_/NF electrode showed higher decay rates of 0.365 and 2.09 mV h^−1^, respectively. Characterizations on NiFe‐LDH@PTFE/NF after the stability test were performed to understand its high stability. The SEM and TEM images shown in Figures S8 and S9 (Supporting Information) indicated negligible morphological changes for NiFe‐LDH@PTFE/NF after the 1000‐h stability test. Conversely, evidence of cracking and catalyst spalling was observed in NiFe‐LDH/NF after just 200 h of use (Figures S10 and S11, Supporting Information), which might be caused by the rapid generation of oxygen bubbles. The Fe content of NiFe‐LDH/NF decreased from 690 to 564 µg cm^−2^ after the stability test for 100 h, while the Fe content of NiFe‐LDH@PTFE/NF remained almost the same (Table S3, Supporting Information). It is demonstrated that PTFE can effectively improve the mechanical stability of OER electrodes and prevent catalyst detachment under high‐current operating conditions. EDS mapping confirmed the persistent uniform distribution of F element in NiFe‐LDH@PTFE/NF after the 1000‐h stability test (Figure S12, Supporting Information), indicating the durability of PTFE during long‐term testing, which is further confirmed by XPS (Figure S13a, Supporting Information). Furthermore, the chemical states of NiFe‐LDH also remained almost unchanged from the original sample (Figure 2h,i and Figure S13b,c, Supporting Information), demonstrating the exceptional chemical stability of NiFe‐LDH@PTFE/NF under oxidation conditions. The outstanding mechanical stability and chemical stability of NiFe‐LDH@PTFE/NF guarantee its sustained high performance, which is crucial for industrial applications.

### 2.3 Theoretical Investigation on Mass Transfer

**Figure 4:**
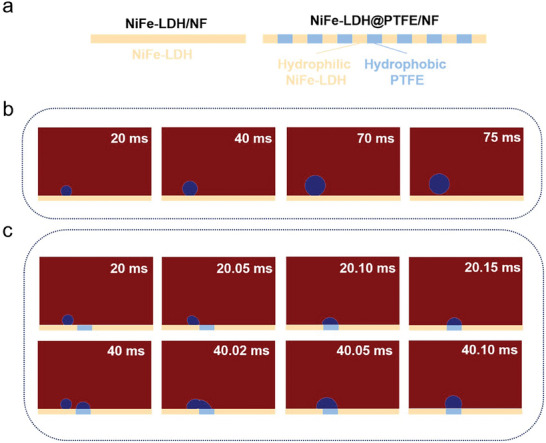
Finite element simulations of bubble evolution on NiFe‐LDH/NF and NiFe‐LDH@PTFE/NF. a) Models of the electrode surface for NiFe‐LDH/NF and NiFe‐LDH@PTFE/NF. b) The evolution of O_2_ bubble on NiFe‐LDH/NF. c) The evolution of O_2_ bubble on NiFe‐LDH@PTFE/NF. The generated O_2_ bubble quickly transferred to the PTFE surface, resulting in enhanced mass transfer around the NiFe‐LDH active sites.

To further understand the synergistic effect of the hydrophobic PTFE and hydrophilic NiFe‐LDH on mass transfer, microfluidic finite element simulations were performed to reveal the gas bubble evolution on NiFe‐LDH/NF and NiFe‐LDH@PTFE/NF. The electrode surface models for NiFe‐LDH/NF and NiFe‐LDH@PTFE/NF are shown in **Figure** **4**a. On the surface of NiFe‐LDH/NF, the O_2_ bubble formed at the NiFe‐LDH active site continues to grow on the NiFe‐LDH surface until it eventually detaches from the electrode surface (Figure 4b), limiting water diffusion around the active sites covered by the O_2_ bubble. The time required for bubble detachment in this model is 75 ms and the diameter of the detached bubble is 35.5 µm. In contrast, for NiFe‐LDH@PTFE/NF, the O_2_ bubble formed at the NiFe‐LDH active site is transferred to the PTFE surface within 0.1 ms (Figure 4c) due to the hydrophobicity of PTFE. Compared to the hydrophilic NiFe‐LDH surface, bubbles are more favorable to adhere to and aggregate on a hydrophobic surface. Although it requires a longer time for bubbles to detach from the surface of NiFe‐LDH@PTFE/NF electrode (with a detached bubble diameter of 101.3 µm), they primarily occupy the PTFE surface rather than NiFe‐LDH active sites. Therefore, the integration of hydrophobic PTFE allows generated O_2_ bubbles to leave NiFe‐LDH active sites quickly and aggregate on PTFE, which is not involved in the electrochemical process, resulting in enhanced mass transfer around the NiFe‐LDH active sites.

### 2.4 Practical Water Electrolysis

**Figure 5:**
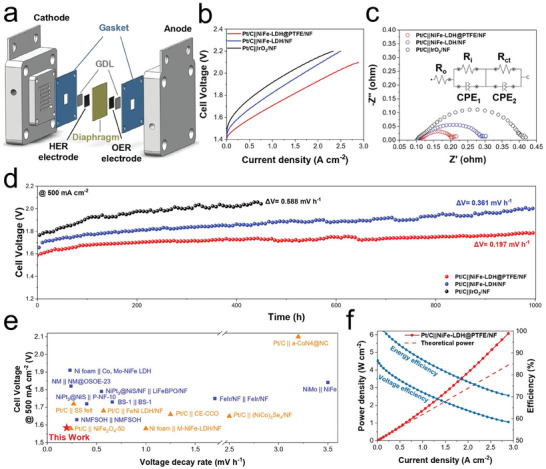
AWE performance under industrial operating conditions. a) Schematic of the AWE cell configuration. b) Polarization curves for AWE using various OER electrodes paired with a standardized commercial Pt/C HER electrode. c) EIS Nyquist plots acquired at 1.6 V. d) Long‐term stability test at a constant current density of 500 mA cm^−2^. e) Comparison of cell voltage at 500 mA cm^−2^ and voltage decay rate during stability test for Pt/C || NiFe‐LDH@PTFE/NF against other reported AWE (blue square) and AEMWE (orange triangle) from previous literature. f) Power density and efficiency as a function of current density for Pt/C || NiFe‐LDH@PTFE/NF. All AWE measurements were conducted at 80 °C with 30 wt% KOH as the electrolyte.

To better evaluate the OER performance of NiFe‐LDH@PTFE/NF under conditions reflective of industrial applications, a zero‐gap AWE was assembled as illustrated in **Figures** **5**a and S14 (Supporting Information). This AWE setup comprised current collectors, gas diffusion layers, electrodes for HER and OER, and a Zirfon UTP 220 separator. The HER electrode was standardized using a commercial Pt/C electrode, as it is one of the widely used HER electrodes in ALK.^[^ ^29^ ^]^ The AWE cell operated at 80 °C using 30 wt% KOH as the electrolyte to mirror industrial conditions closely.^[^ ^7a^ ^]^ When evaluated with the identical HER electrode, NiFe‐LDH@PTFE/NF demonstrated a much better performance than NiFe‐LDH/NF and the commercial IrO_2_/NF electrode. Specifically, cell voltages for Pt/C || NiFe‐LDH@PTFE/NF were recorded at 1.584, 1.706, and 1.928 V for current densities of 0.5, 1.0, and 2.0 A cm^−2^, respectively (Figure 5b). These results not only surpass those of previously reported AWE but are also competitive with some anion exchange membrane water electrolyzers (AEMWE) shown in Table S4 (Supporting Information). In comparison, Pt/C || NiFe‐LDH/NF required higher voltages of 1.676, 1.821, and 2.079 V for the same current densities. It is noted that the voltage differential became larger as the current increased, demonstrating the enhanced mass transfer capabilities afforded by the hydrophobic–hydrophilic synergy of PTFE and NiFe‐LDH. EIS further substantiated these findings, with parameters of fitted Nyquist plots summarized in Table S5 (Supporting Information). The equivalent circuit model (inset of Figure 5c) identified *R*_o_ as the ohmic resistance within the AWE system, *R*_i_ as the ion transfer impedance in high‐frequency region, and *R*_ct_ as the charge transfer impedance of both HER and OER process in mid‐high frequency region.^[^ ^30^ ^]^ The subsequent low‐frequency tail beyond the *R*_ct_ semicircle was attributed to the mass transfer impedance. The *R*_o_ remained consistent across different AWE cells, which suggests the interference of system resistance brought by cell assembly was negligible when comparing their performance. The Pt/C || NiFe‐LDH@PTFE/NF exhibited a significantly reduced *R*_ct_ (0.090 Ω) compared to Pt/C || NiFe‐LDH/NF (0.184 Ω) and Pt/C || IrO_2_/NF (0.282 Ω), evidencing the rapid OER kinetics facilitated by NiFe‐LDH@PTFE/NF. Remarkably, Pt/C || NiFe‐LDH@PTFE/NF maintained excellent stability over 1000 h at a current density of 500 mA cm^−2^ with a minimal potential decay rate of 0.197 mV h^−1^ (Figure 5d), attributed to the high alkali resistance of PTFE in severe conditions.^[^ ^31^ ^]^ In contrast, the potential decay rate increased to 0.361 and 0.588 mV h^−1^ when the OER electrode was replaced with NiFe‐LDH/NF and commercial IrO_2_/NF, respectively. Although it is difficult to quantify the individual contribution of HER electrode, OER electrode, and other components of AWE to cell performance degradation, this control experiment still demonstrates the substantial stability enhancement of the OER electrode through PTFE integration. Figure 5e and Table S4 (Supporting Information) compared the cell performance and the stability of Pt/C || NiFe‐LDH@PTFE/NF with previously reported AWE or AEMWE, demonstrating the superior performance and stability of NiFe‐LDH@PTFE/NF under industrial conditions.

The Pt/C || NiFe‐LDH@PTFE/NF present in this work achieves an impressive high energy efficiency of 93.5% for water electrolysis at a current density of 500 mA cm^−2^, as shown in Figure 5f (see Note in the Supporting Information for detailed calculation). Moreover, the durable NiFe‐LDH@PTFE/NF electrode ensures the electrolyzer sustains high energy efficiency over extended periods, thereby reducing the cost associated with catalyst replacement during continuous operation. The combination of superior energy efficiency and reduced maintenance costs positions NiFe‐LDH@PTFE/NF as a highly promising candidate for industrial‐scale water electrolysis applications.

## 3 Conclusion

The in situ integration of PTFE can significantly enhance the activity and stability of OER electrodes is demonstrated in this work using NiFe‐LDH electrocatalyst as a proof of concept. It is important to note that the catalyst itself still has substantial potential for further optimization. As PTFE does not directly participate in the electrochemical process but rather achieves performance enhancement by stabilizing the electrocatalyst and improving the mass transfer toward water oxidation, it can be incorporated into electrocatalysts with more advanced catalytic mechanisms to further elevate the overall performance. Beyond OER, this methodology holds promise for refining electrode design across a spectrum of electrochemical processes, including HER, ORR, and CO_2_RR, where enhancing stability and mass transfer are universally sought objectives.

In summary, we have presented an innovative strategy of incorporating PTFE as a multifunctional binder into OER electrodes to obtain a more durable electrode with better mass transfer toward OER. A NiFe‐LDH@PTFE/NF electrode was chosen for the proof of concept, which displayed an incredibly low potential decay rate of 0.034 mV h^−1^ at a high current density of 500 mA cm^−2^. Furthermore, when combined with commercial Pt/C as the HER electrode, the AWE using NiFe‐LDH@PTFE/NF for OER required only 1.584 V at 500 mA cm^−2^ and exhibited remarkable stability under industrial operating conditions. This work not only introduces a novel concept for the employment of PTFE or similar multifunctional binders in enhancing OER electrode performance but also lays down a versatile strategy for electrode design across various electrochemical reactions, bringing great advances to other renewable energy technologies.

## 4 Experimental Section

### Chemicals and Materials

Nickel chloride hexahydrate (NiCl_2_·6H_2_O, AR, >99%), ferrous chloride tetrahydrate (FeCl_2_·4H_2_O, GR, 99.95% metals basis), and potassium hydroxide (KOH, 99.99% metals basis, except sodium) were purchased from Macklin. PTFE preparation (60 wt% dispersion in H_2_O) was purchased from Shenzhen Kejing Star Technology Co. Ltd. Acetone (CH_3_COCH_3_, AR, >99.5%) was purchased from Aladdin. Potassium chloride (KCl, GR, >99.8%) and hydrochloric acid (HCl, 37%) were purchased from Sinopharm. All chemicals were used directly without any purification. Nickel foam (1 mm thick) was purchased from Lizhiyuan Co. Ltd. Nickel felt (0.5 mm thick), commercial Pt/C electrodes (1.0 mg cm^−2^), and commercial IrO_2_/NF electrodes (2.5 mg cm^−2^) were purchased from Sinero Technology Ltd.

### Preparation of NiFe‐LDH/NF and NiFe‐LDH@PTFE/NF Electrodes

The NiFe‐LDH/NF and NiFe‐LDH@PTFE/NF electrodes were prepared using an electroplating method. Before electroplating, Ni foam substrates were cut into 1 cm × 1.5 cm pieces and thoroughly washed in acetone and 1 m hydrochloric acid for 15 min by ultrasonication to remove impurities and the surface oxide layer, then washed with deionized water.

A mixed solution of 0.075 m NiCl_2_·6H_2_O, 0.025 m FeCl_2_·4H_2_O, and 1 m KCl dissolved in deionized water was used as the electroplating solution to prepare NiFe‐LDH/NF electrodes. 60 wt% PTFE dispersion was added into the electroplating solution described above in the preparation of NiFe‐LDH@PTFE/NF electrodes. The added amount of PTFE was 10 g L^−1^ unless otherwise noted. Each Ni foam substrate was dipped into the electroplating solution with a plating area of 1 cm × 1 cm and the remaining part was used as the electrical contact. The electrochemical deposition was performed in a three‐electrode system, in which a Ni foam electrode and an Ag/AgCl reference electrode were used as the counter electrode and the reference electrode, respectively. The deposition was conducted via chronoamperometry mode with a cathodic potential of −1.1 V versus Ag/AgCl for 5 min. After deposition, the electrodes were washed thoroughly with deionized water, then dried in air overnight.

### Characterizations

SEM images were taken by a Zeiss microscope (MERLIN VP Compact) operated at 15 kV. The FTIR spectra were collected using an FTIR spectrometer (iS50, Nicolet). Raman spectra were collected using a confocal Raman microscope (LabRAM HR Evolution, HORIBA Jobin Yvon) with a wavelength of 532 nm and a power of 5 mW at the objective. X‐ray diffraction analysis was performed by a D/max 2500 V diffractometer in reflection mode at 40 kV and 150 mA with a scanning speed of 8° min^−1^. X‐ray photoelectron spectra were collected using a Thermo Fisher spectrometer (Escalab 250Xi) equipped with an Al Kα radiation source (1487.6 eV) and hemispherical analyzer with a pass energy of 30.0 eV and energy step size of 0.05 eV. All the XPS spectra were corrected by C 1s peak of 284.8 eV and fitted using XPSPEAK41 software with Shirley backgrounds and Gaussian–Lorentzian functions. TEM and HRTEM images were taken by a 2100F transmission electron microscope operated at 200 kV. The Fe content of electrodes was measured by an inductively coupled plasma optical emission spectrometer (IRIS Intrepid II XSP, Thermo Fisher). N_2_ adsorption–desorption isotherms were determined at 77 K using a specific surface and pore size analyzer (Quadrasorb SI‐MP) and the pore size distributions were obtained via the Barrett–Joyner–Halenda (BJH) method.

### Electrochemical Measurement

All electrochemical tests were carried out on an electrochemical workstation (PGSTAT204, Autolab). The OER performance of working electrodes was measured at 25 °C in a three‐electrode system, in which a graphite rod and an Hg/HgO reference electrode were used as the counter electrode and the reference electrode, respectively. For three‐electrode measurements, the geometric area of the working electrode was 0.5 × 1.0 cm^2^ and the electrolyte was 1 m KOH solution. The measured potential versus Hg/HgO reference electrode in 1 m KOH solution could be converted to the potential versus reversible hydrogen electrode (RHE) by the following equation

(1) $$E_{\mathrm{RHE}}=E_{\mathrm{Hg}/\mathrm{HgO}}+0.926\;V$$

The CV curves were collected at a slow scan rate of 1 mV s^−1^ to exclude the interference of the Ni^2+^/Ni^3+^ oxidation current on the evaluation of electrocatalytic activity toward OER. IR corrected by 90% of the solution resistance was used to eliminate the effect of solution resistance. The resistance was measured by EIS with high frequency of 100 kHz and low frequency of 0.01 Hz at 0.6 V versus Hg/HgO. The ECSA was fitted by a series of cyclic voltammetry curves at different scan rates varying from 1 to 100 mV s^−1^ in the potential range of −0.3 to −0.2 V versus Hg/HgO (where no Faradaic reaction occurred). For the stability test, chronopotentiometry was applied at a current density of 500 mA cm^−2^.

### Finite Element Simulations

Microfluid finite element models were established using a two‐phase flow simulation method. For the sake of simplicity, the dynamic behavior of bubble generation and evolution was analyzed in a two‐dimensional flow field. The fluid region size in two‐phase flow had a length of 200 µm and a height of 120 µm. The coordinate range of the fluid region in the horizontal direction is −50 to 150 µm. The left side of the fluid domain was set as the inlet with a flow rate of 2 × 10^−7^ m s^−1^, while the top and right sides of the fluid domain were set as outlets. For NiFe‐LDH/NF, the electrode surface was modeled as a homogeneous hydrophilic surface representing NiFe‐LDH. For NiFe‐LDH@PTFE/NF, the electrode surface consisted of both hydrophilic and hydrophobic surfaces, representing NiFe‐LDH and PTFE, respectively. The contact angles of the hydrophilic and hydrophobic surfaces were set as 18° and 130°, respectively. The oxygen bubbles were generated on the hydrophilic NiFe‐LDH surface at a generated rate of 450 µm^3^ ms^−1^.

The process of gas–liquid interface evolution was calculated by the following equations

(2) $$\rho \frac{\partial u}{\partial t}+\rho \left(u\;\nabla \right)u=\nabla \left[-pI+\mu \left(\nabla u+\nabla u^{T}\right)\right]+F_{\mathrm{st}}+F$$

(3) ∇u=0

where ρ is the fluid density, *u* is the fluid velocity, *F* is the volume force, and *F*_st_ indicates the surface tension acting at the interface between the two fluids which can be expressed as

(4) $$F_{\mathrm{st}}=\;\sigma \delta \kappa n+\delta \nabla_{s}\sigma$$

where σ is the surface tension coefficient (N m^−1^), δ (1 m^−1^) is a Dirac delta function on the interface and κ  =   − ∇ *n* is the curvature, *n* is the unit normal to the interface, and ∇_*s*_ is the surface gradient operator.

### Alkaline Water Electrolyzer Measurement

The overall performance of water splitting was evaluated using an AWE shown in Figure 5a and Figure S14 (Supporting Information). The Pt/C electrodes with a platinum loading of 1.0 mg cm^−2^ were used as HER electrodes. The NiFe‐LDH/NF, NiFe‐LDH@PTFE/NF, and commercial IrO_2_/NF electrodes with an IrO_2_ loading of 2.5 mg cm^−2^ were used as OER electrodes respectively for comparison. The active area of both HER and OER electrodes was 1.0 × 1.0 cm^2^. The separator and gas diffusion layer used in the AWE cell were Zirfon UTP 220 and nickel felt, respectively.

All the AWE measurements were performed on an electrochemical workstation (EnergyLab XM + External Booster, Solartron Analytical). The cell temperature was maintained at 80 °C under atmospheric pressure and preheated electrolyte (30 wt% KOH) was supplied to both sides at a flow rate of 10 mL min^−1^ during the test. The AWE cell was first activated by conducting 20‐cycle CV ranging from 1.2 to 2.2 V with a scan rate of 10 mV s^−1^. After the activation process, the AWE performance was evaluated via the voltage sweep method from 1.2 to 2.2 V with a scan rate of 10 mV s^−1^. EIS was performed at a constant voltage of 1.6 V with an amplitude of 10 mV and frequency ranging from 10 kHz to 0.1 Hz. The durability of the AWE was evaluated via chronopotentiometry at a current density of 500 mA cm^−2^.

## Conflict of Interest

The authors declare no conflict of interest.

## Supporting information

Supporting Information

## Data Availability

The data that support the findings of this study are available from the corresponding author upon reasonable request.
