# Supplementary material for: PTFE as a Multifunctional Binder for High‐Current‐Density Oxygen Evolution
Source: Adv Sci (Weinh). 2024 Sep 4;11(41):2408544. doi: 10.1002/advs.202408544 (PMC11538630; doi:10.1002/advs.202408544)
Supplement: Supplementary file 1 — Supporting Information [file ADVS-11-2408544-s001.docx]

Supporting Information

PTFE as a Multifunctional Binder for High-Current-Density Oxygen Evolution

Bohan Deng, Xian He, Peng Du, Wei Zhao, Yuanzheng Long, Zhuting Zhang, Hongyi Liu, Kai Huang*, and Hui Wu*

Bohan Deng, Wei Zhao, Yuanzheng Long, Zhuting Zhang, Hongyi Liu, Hui Wu

State Key Lab of New Ceramics and Fine Processing, School of Materials Science and Engineering, Tsinghua University, Beijing, 100084, China
E-mail: huiwu@tsinghua.edu.cn

Xian He, Peng Du, Kai Huang
State Key Laboratory of Information Photonics and Optical Communications & School of Science, Beijing University of Posts and Telecommunications, Beijing, 100876, China

E-mail: huang-kai@bupt.edu.cn

Hongyi Liu,

Dongfang Electric (Fujian) Innovation Research Institute Co., Ltd, Fujian Province, 350108, China

1. **Supplementary Note: Calculations on the overall energy efficiency**

The energy required for hydrogen production equals the enthalpy change (ΔH) of water splitting reaction, while the minimum electricity required for hydrogen production equals the Gibbs free energy change (ΔG) of water splitting reaction.

When T = 298.15 K, ΔH^0^ = 285.83 kJ mol^-1^, and ΔG^0^ = 237.14 kJ mol^-1^. Therefore, the energy efficiency is calculated as:

$$\eta_{e}=\frac{{\Delta H}^{0}\cdot\frac{It}{nF}}{UIt}=\frac{{\Delta H}^{0}}{UnF}=\frac{285.83 kJ\cdot{mol}^{-1}}{U\cdot2\cdot96485 C\cdot{mol}^{-1}}=\frac{1.481 V}{U}$$

while the voltage efficiency is calculated as:

$$\eta_{v}=\frac{{\Delta G}^{0}\cdot\frac{It}{nF}}{UIt}=\frac{{\Delta G}^{0}}{UnF}=\frac{237.14 kJ\cdot{mol}^{-1}}{U\cdot2\cdot96485 C\cdot{mol}^{-1}}=\frac{1.229 V}{U}$$

The cell voltages at 500 mA cm^-2^ for Pt/C || NiFe-LDH@PTFE/NF was 1.584 V, so the corresponding energy efficiency η_e_= 1.481/1.584*100% = 93.5%.

1. **Supplementary Figures:**


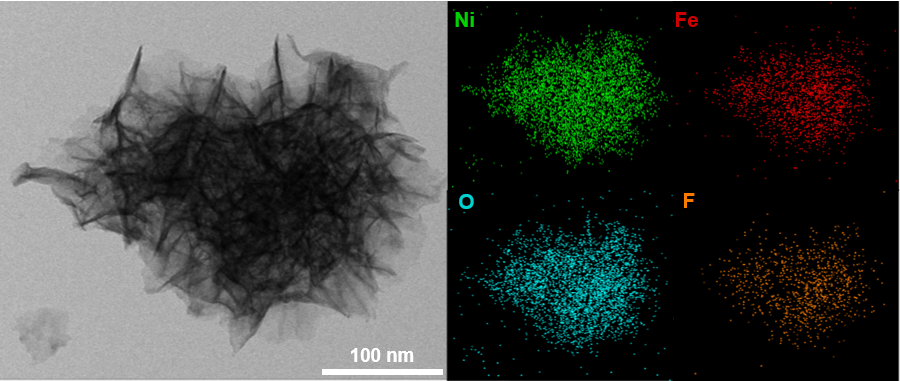


**Figure S1.** TEM image of NiFe-LDH@PTFE/NF and corresponding EDS elemental mapping of Ni, Fe, O, and F.


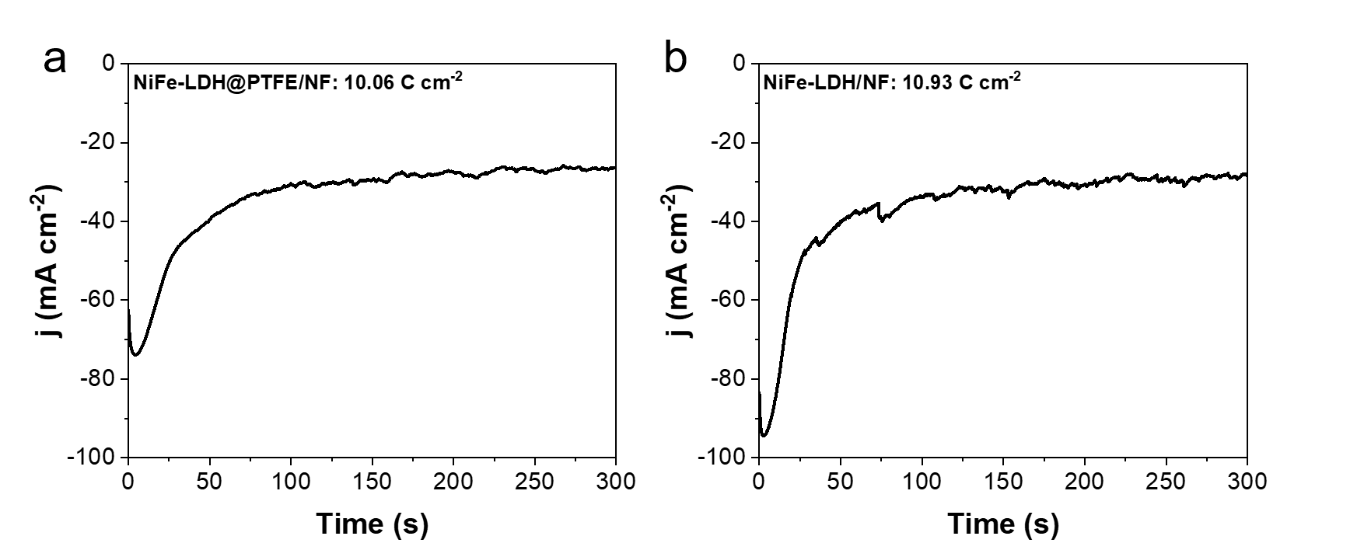


**Figure S2.** I-t profiles during the electroplating process in the fabrication of (a) NiFe-LDH@PTFE/NF and (b) NiFe-LDH/NF. The lower electroplating current in the fabrication of NiFe-LDH@PTFE/NF is attributed to the decrease in conductivity of the electroplating solution with the addition of PTFE.

**
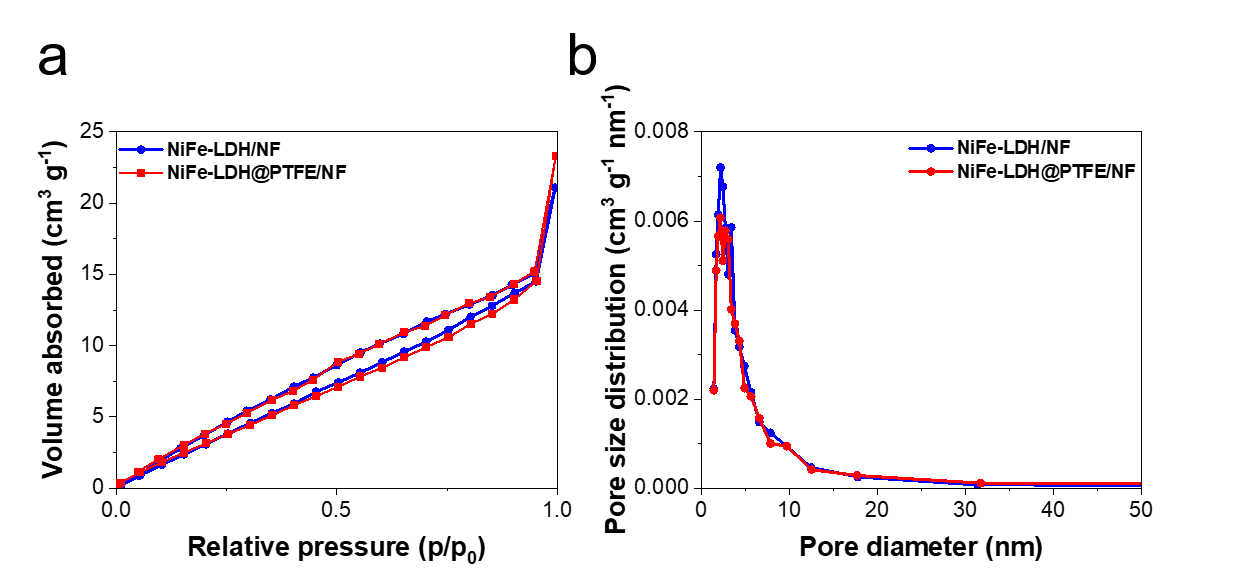
**

**Figure S3.** (a) N_2_ adsorption-desorption isotherm curves and (b) pore size distributions of NiFe-LDH/NF and NiFe-LDH@PTFE/NF


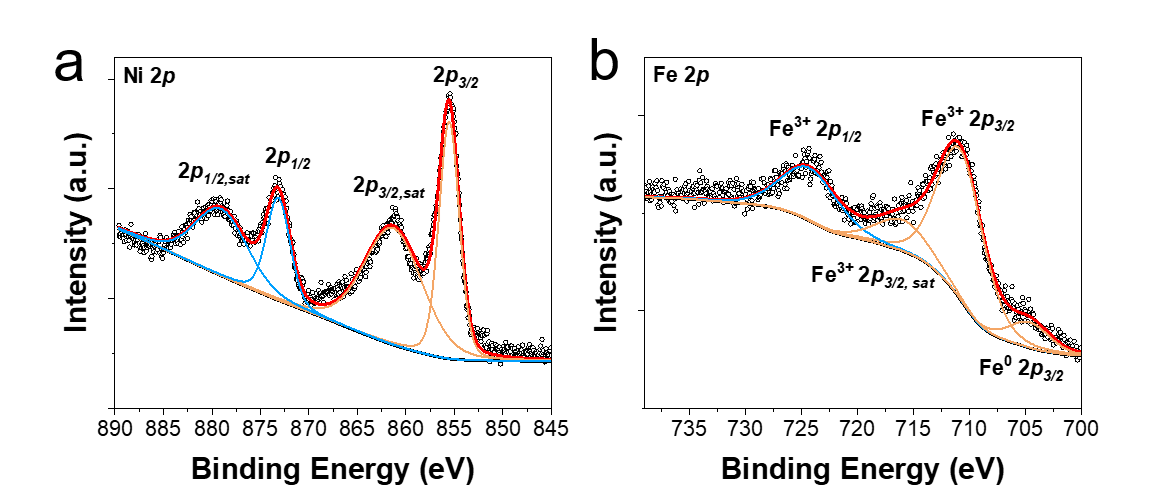


**Figure S4.** XPS spectra of NiFe-LDH/NF. (a) Ni 2*p* (b) Fe 2*p*.


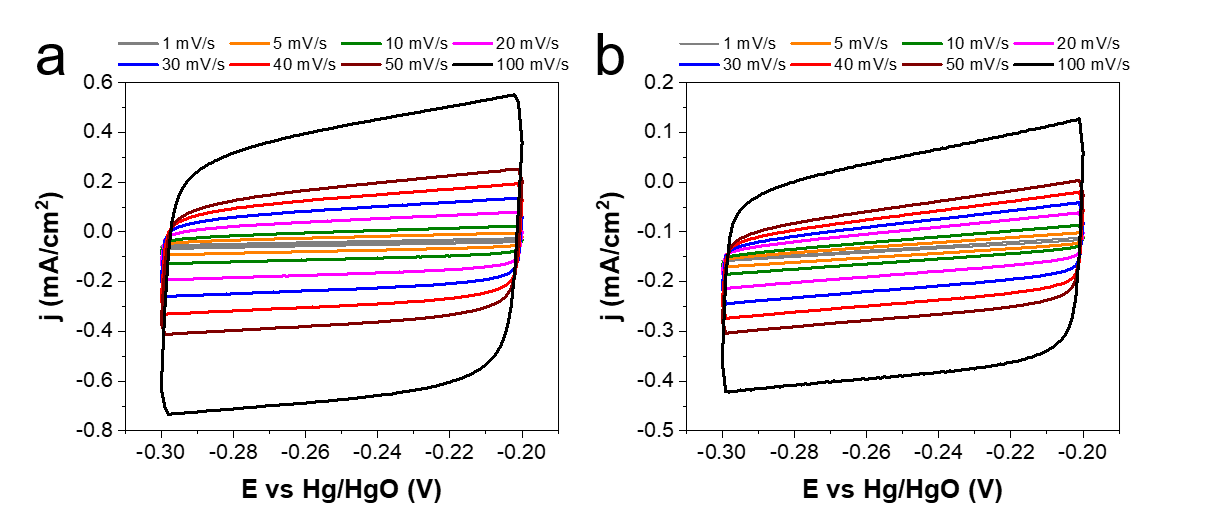


**Figure S5.** Cyclic voltammetry polarization curves collected in the non-faradaic potential (-0.3 ~ -0.2 V vs Hg/HgO) at various scan rates for (a) NiFe-LDH@PTFE/NF (b) NiFe-LDH/NF.


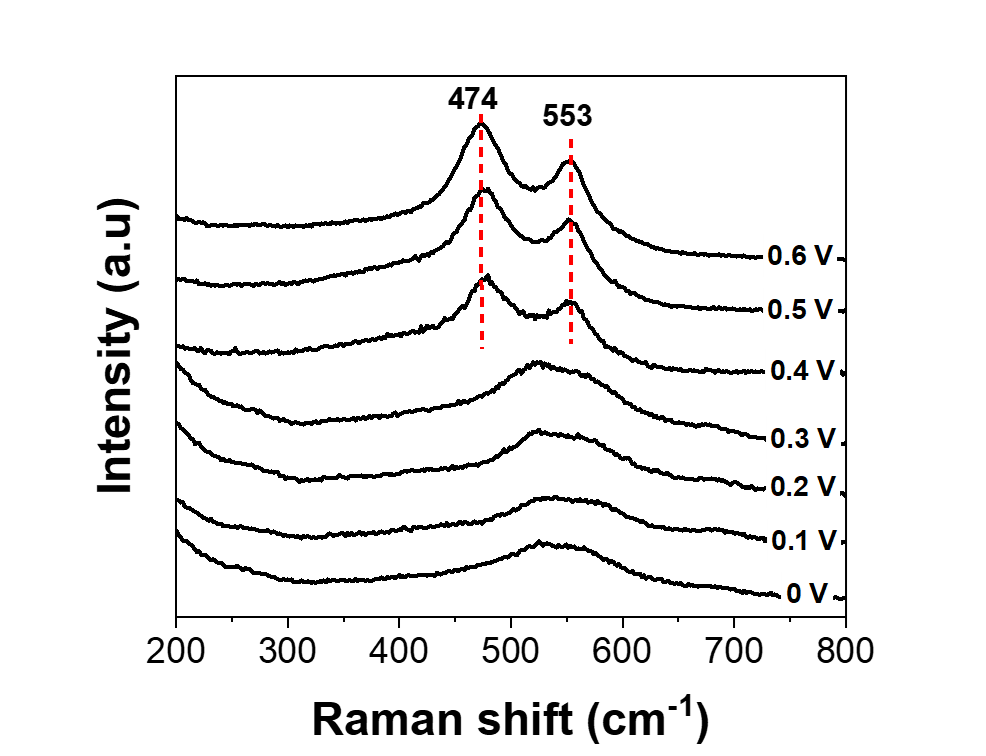


**Figure S6.** In situ Raman spectra of NiFe-LDH@PTFE/NF electrode as a function of potential vs Hg/HgO.


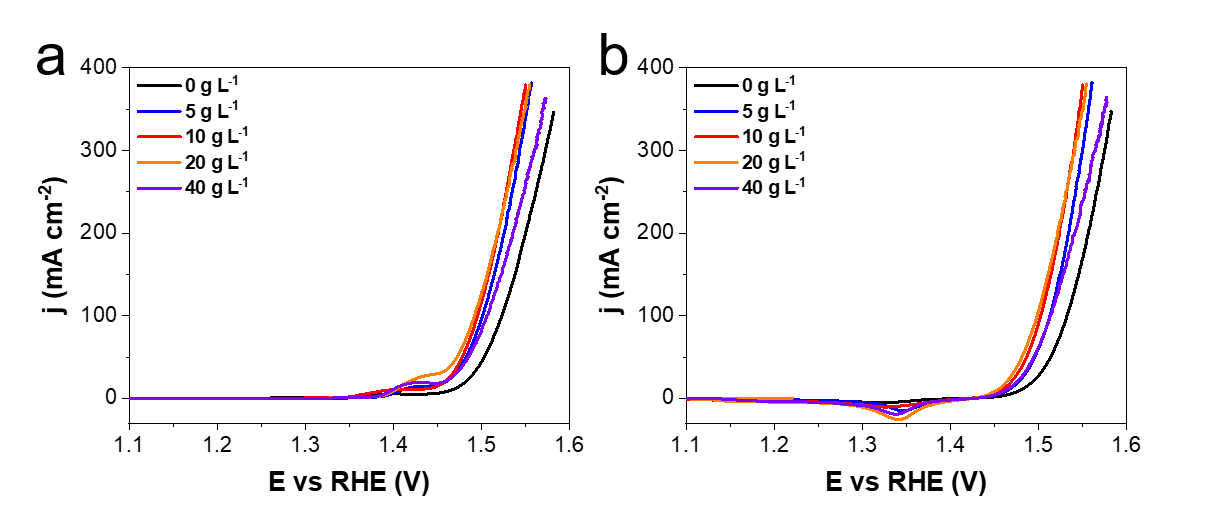


**Figure S7.** LSV polarization curves for NiFe-LDH@PTFE/NF with various PTFE concentrations in the electroplating solution. (a) ascending scan from lower to higher potential. (b) descending scan from higher to lower potential.


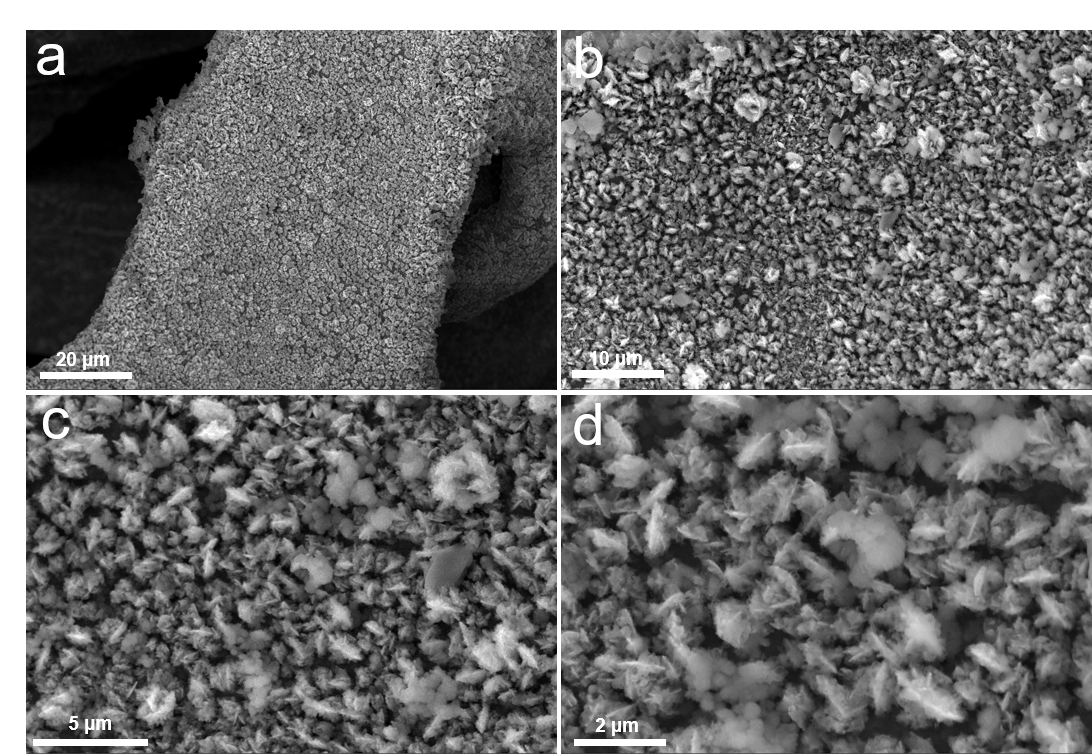


**Figure S8.** SEM images of NiFe-LDH@PTFE/NF after stability test (chrono-potentiometry at 500 mA cm^−2^ for 1000 hours) at different magnifications.


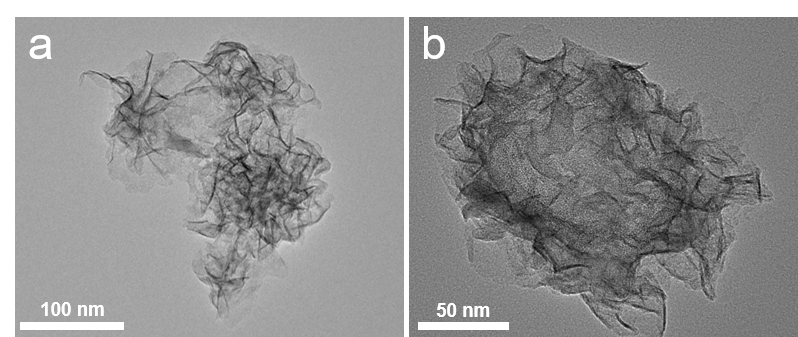


**Figure S9.** TEM images of NiFe-LDH@PTFE/NF after stability test (chrono-potentiometry at 500 mA cm^−2^ for 1000 hours) at different magnifications.


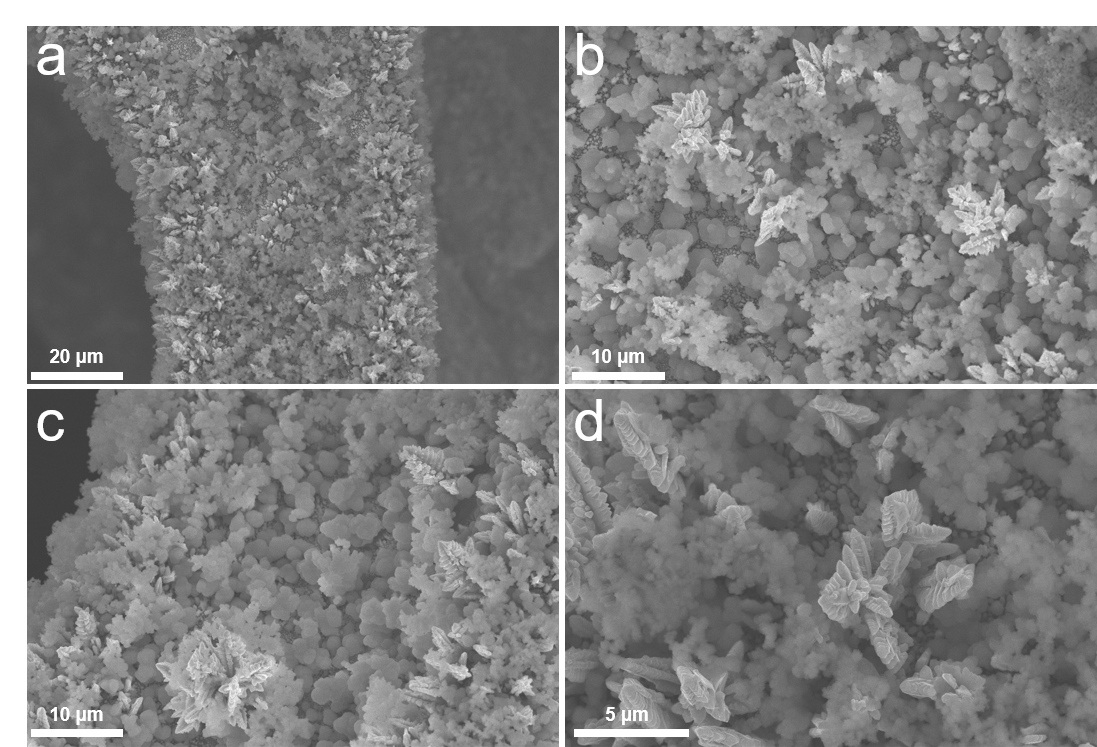


**Figure S10.** SEM images of NiFe-LDH/NF at different magnifications.


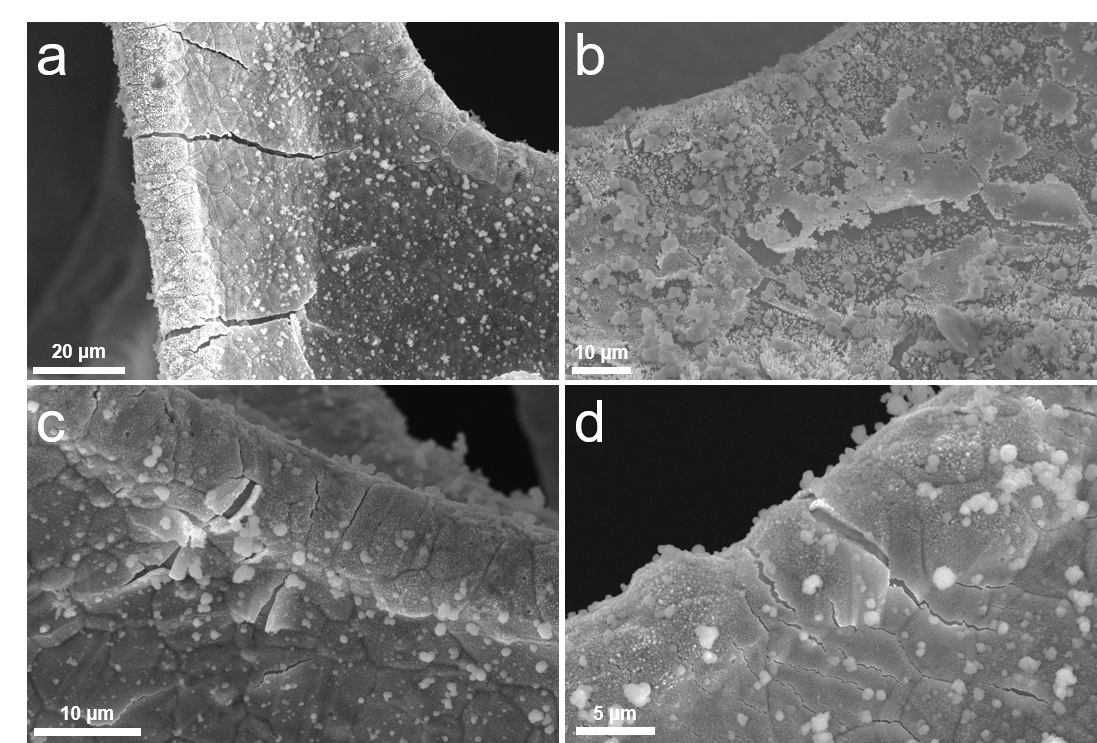


**Figure S11.** SEM images of NiFe-LDH /NF after stability test (chronopotentiometry at 500 mA cm^−2^ for 200 hours) at different magnifications.


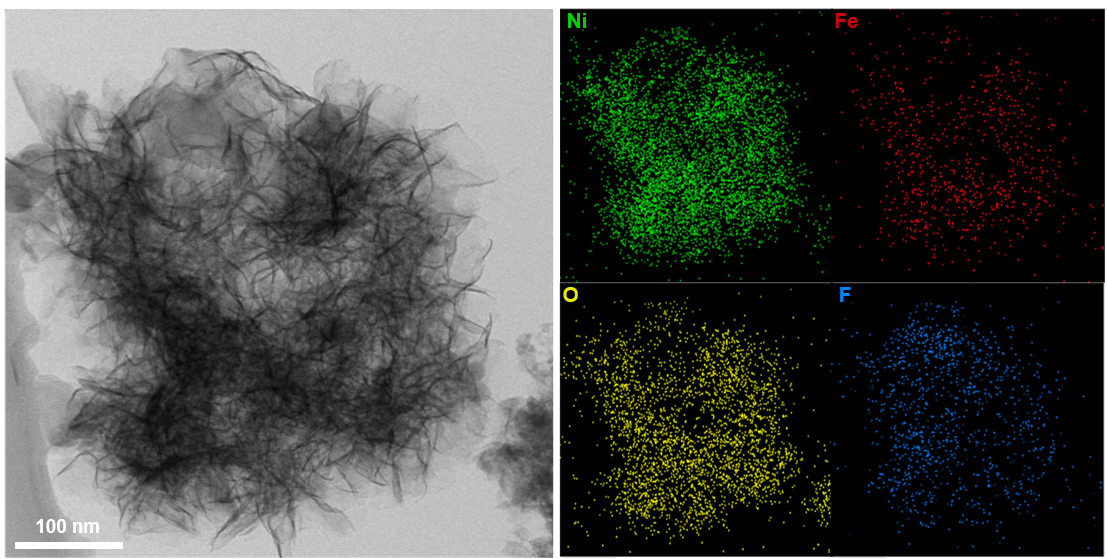


**Figure S12.** TEM image of NiFe-LDH@PTFE/NF after the stability test and corresponding EDS elemental mapping of Ni, Fe, O, and F.


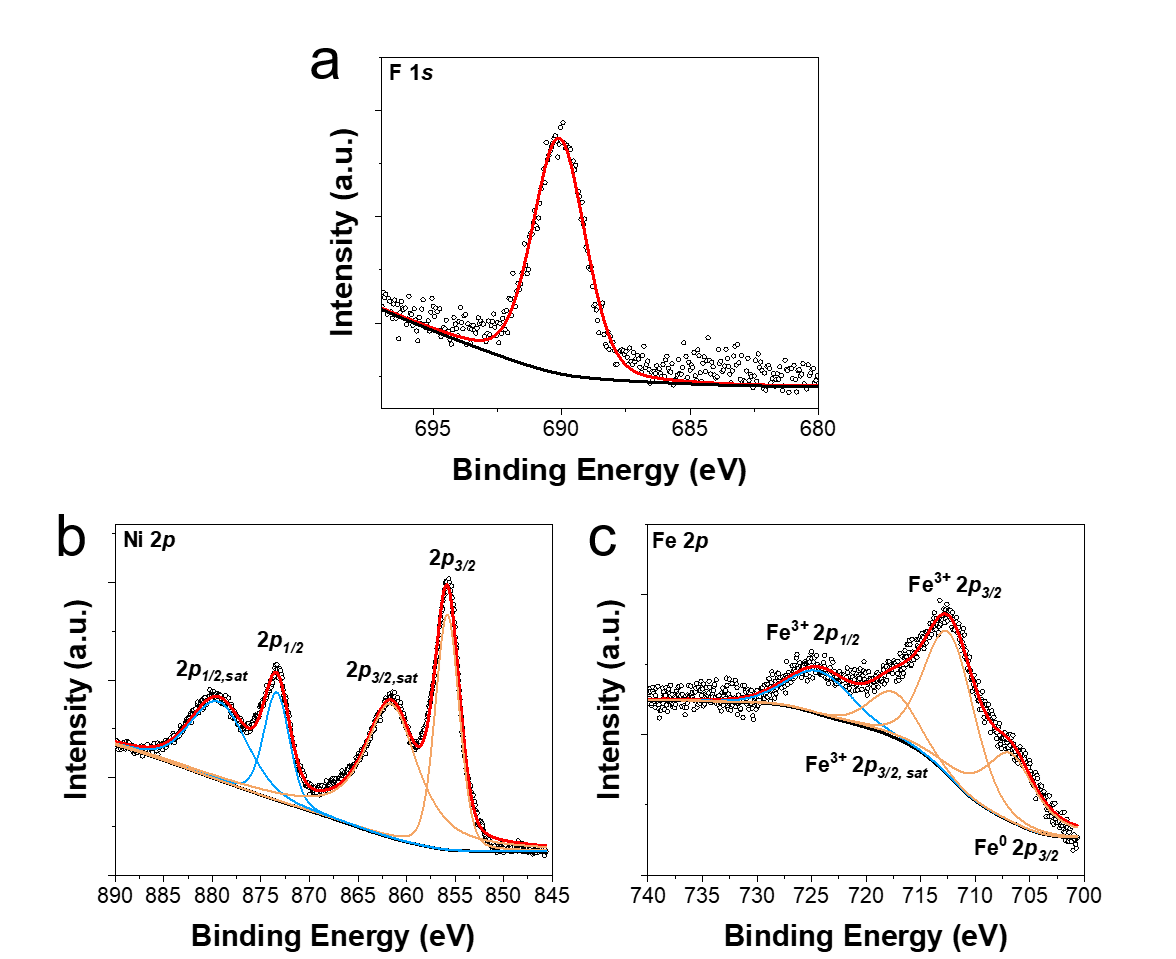


**Figure S13.** XPS spectra of NiFe-LDH@PTFE/NF after the stability test. (a) F 1*s* (b) Ni 2*p* (c) Fe 2*p*.


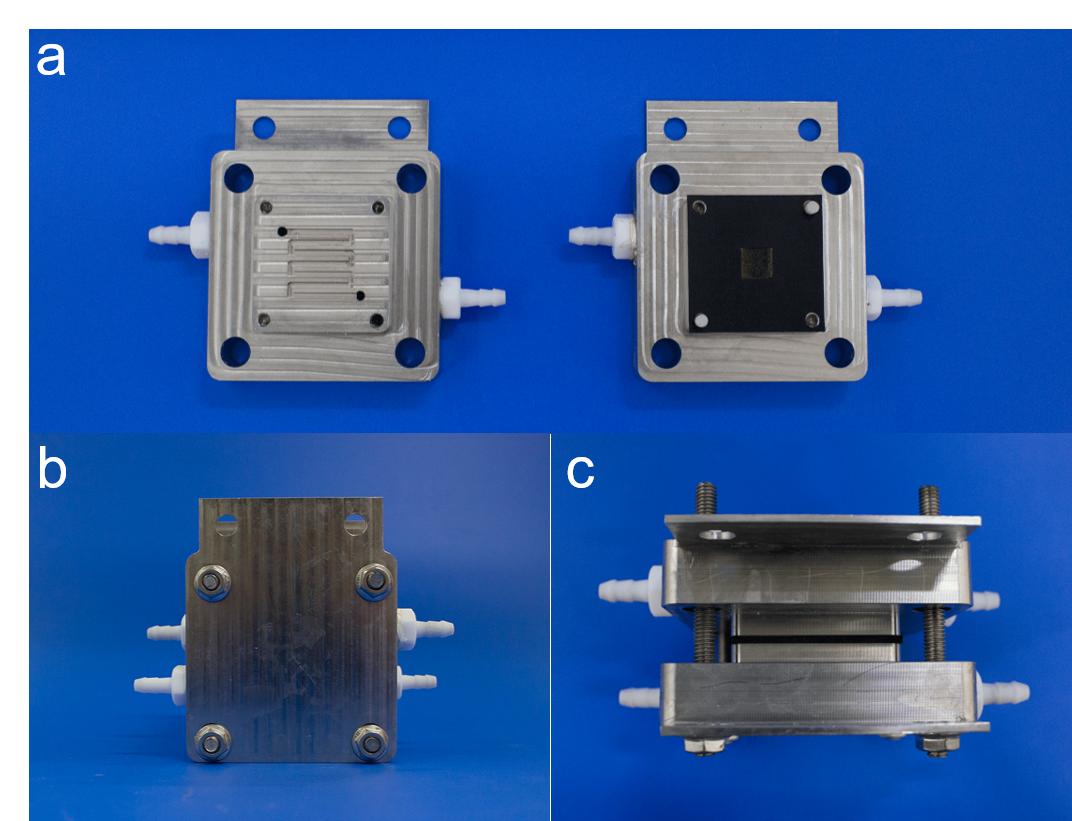


**Figure S14.** Optical photographs of the applied AWE cell.

1. **Supplementary Tables:**

**Table S1.** Optimum fit parameters of the EIS data in Figure 3e to the equivalent circuit.

| Sample | R_s_ (Ω) | R_f_ (Ω) | R_ct_ (Ω) |
| --- | --- | --- | --- |
| NiFe-LDH@PTFE/NF | 1.68 | 0.049 | 0.526 |
| NiFe-LDH/NF | 1.95 | 0.072 | 0.851 |
| commercial IrO_2_/NF | 1.96 | 0.091 | 2.64 |

**Table S2.** Comparison of the NiFe-LDH@PTFE/NF with other reported OER catalysts in the performance towards water oxidation.

| Catalyst | Substrate | η (mV) @  10 mA cm^-2^ | η (mV) @  100 mA cm^-2^ | Tafel slope  (mV dec^-1^) | Stability | Reference | |
| --- | --- | --- | --- | --- | --- | --- | --- |
| NiFe-LDH@PTFE | Ni Foam | 228 | 273 | 36.6 | 1000h@500 mA cm^-2^ | This work |  |
| NiFe-LDH | Ni Foam | 251 | 303 | 44.3 | / | This work |  |
| NiFe-LDH | Ni Foam | 246 | / | 46.6 | 20h@~40 mA cm^-2^ | ^[1]^ |  |
| NiFe LDH/NiS | Ni Foam | 230 | 277 | 60.1 | 40h@400 mA cm^-2^ | ^[2]^ |  |
| NiFe LDH-POM | Ni Foam | ~210 | ~310 | 67 | 20h@100 mA cm^-2^ | ^[3]^ |  |
| NiFe LDH@NiCoP | Ni Foam | ~230 | ~350 | 48.6 | 100h@10 mA cm^-2^ | ^[4]^ |  |
| NiFe-LDH | Glassy carbon | 270 | / | 112 | / | ^[5]^ |  |
| d-NiFe-LDH | Glassy carbon | 230 | ~340 | 77 | 36h@10 mA cm^-2^ | ^[5]^ |  |
| NiFeCo-LDH/CF | Glassy carbon | 249 | ~305 | 42 | 10h@10 mA cm^-2^ | ^[6]^ |  |
| MoNiFe-27%  (oxy)hydroxide | Cabron cloth | 242 | 290 | 23 | 20h@100 mA cm^-2^ | ^[7]^ |  |
| Ni/Ni(OH)_2_ | Carbon paper | 270 | ~340 | 70 | 20h@10 mA cm^-2^ | ^[8]^ |  |
| Ir_18 wt %_-NiO | Cabron cloth | 215 | ~255 | 38 | 10h@10 mA cm^-2^ | ^[9]^ |  |
| a-NiCo/NC | Carbon paper | 252 | ~310 | 49 | 150h@10 mA cm^-2^ | ^[10]^ |  |

**Table S3.** Fe content of NiFe-LDH@PTFE/NF and NiFe-LDH/NF before and after electrochemical measurements measured by ICP-OES.

| Sample | Fe content (μg cm^-2^) |
| --- | --- |
| NiFe-LDH/NF | 690 |
| NiFe-LDH@PTFE/NF | 619 |
| NiFe-LDH/NF after testing for 100h | 564 |
| NiFe-LDH@PTFE/NF after testing for 100h | 621 |

**Table S4.** Comparison of the Pt/C || NiFe-LDH@PTFE/NF with other reported AWE and AEMWE cells in the performance towards overall water-splitting.

| Electrodes  (HER \|\| OER) | Electrolyzer | Operating Temperature & Electrolyte | Current density  (A cm^-2^) | Cell voltage  (V) | Testing time (h) | | Voltage decay rate  (mV h^-1^) | Ref. |
| --- | --- | --- | --- | --- | --- | --- | --- | --- |
| Pt/C \|\| NiFe-LDH@PTFE/NF | AWE | 80 °C 30% KOH | 0.5 | 1.584 | 1000 | 0.197 | | This work |
|  |  |  | 1.0 | 1.706 |  |  | |  |
|  |  |  | 2.0 | 1.928 |  |  | |  |
| NiPt_3_@NiS/NF \|\| LiFeBPO/NF | AWE | 85 °C 6 M KOH | 0.5 | 1.79 | 330 | ~0.55 | | ^[11]^ |
| NiMo \|\| NiFe | AWE | 80 °C 30% KOH | 0.5 | 1.841 | 50 | ~3.5 | | ^[12]^ |
| BS-1 \|\| BS-1 (CoNi phosphide/oxide) | AWE | 85 °C 6 M KOH | 0.5 | ~1.73 |  |  | | ^[13]^ |
|  |  |  | 2.0 | 1.98 | 330 | 0.66 | |  |
| NMFSOH \|\| NMFSOH (Ni–Mo–Fe ternary metal hydroxysulfide) | AWE | 80 °C 6M KOH | 0.5 | ~1.63 | 300 | ~0.3 | | ^[14]^ |
|  |  |  | 1.0 | 1.73 |  |  | |  |
| NM \|\| NM@OSOE-23 | AWE | 70 °C 6M KOH | 0.5 | ~1.82 |  |  | | ^[15]^ |
|  |  |  | 1.0 | ~1.91 | 150 | ~0.24 | |  |
| Ni foam \|\| Co, Mo-NiFe LDH | AWE | 85 °C 30% KOH | 0.4 | 1.91 | 100 | ~0.23 | | ^[16]^ |
| NiPt_3_@NiS \|\| P-NF-10 | AWE | 60 °C 6M KOH | 0.5 | 1.72 | 107 | ~0.4 | | ^[17]^ |
| FeIr/NF \|\| FeIr/NF | AWE | 60 °C 30% KOH | 0.5 | 1.75 | 100 | 1.7 | | ^[18]^ |
| Pt/C \|\| FeNi LDH/NF (IE 20 wt.%) | AEMWE | 60 °C pure water | 0.5 | ~1.68 | 175 | 0.57 | | ^[19]^ |
| Pt/C \|\| NiFe_2_O_4_-50 | AEMWE | 80 °C 1M KOH | 0.5 | ~1.58 |  |  | | ^[20]^ |
|  |  |  | 1.0 | 1.66 | 1000 | 0.24 | |  |
| Pt/C \|\| (NiCo)_3_Se_4_/NF | AEMWE | 60 °C 1M KOH | 0.5 | ~1.65 |  |  | | ^[21]^ |
|  |  | 25 °C 1M KOH | 1.0 | ~1.88 | 95 | ~2.5 | |  |
| Pt/C \|\| a-CoN4@NC | AEMWE | 80 °C 1 M KOH | 0.1 | 1.7 | 35 | ~3.2 | | ^[22]^ |
|  |  |  | 0.5 | 2.1 |  |  | |  |
| Pt/C \|\| CE-CCO | AEMWE | 45 °C 1 M KOH | 0.5 | 1.66 | 64 | 1.25 | | ^[23]^ |
| Pt/C \|\| SS felt | AEMWE | 60 °C 1M KOH | 0.6 | 1.72 | 100 | 0.268 | | ^[24]^ |
| Ni foam \|\| M-NiFe-LDH/NF | AEMWE | 50 °C 1M KOH | 0.5 | ~1.58 |  |  | | ^[25]^ |
|  |  |  | 1.0 | 1.69 | 50 | ~1 | |  |

**Table S5.** Optimum fit parameters of the EIS data in Figure 5c to the equivalent circuit.

| AWE cell | R_o_ (Ω) | R_i_ (Ω) | R_ct_ (Ω) |
| --- | --- | --- | --- |
| Pt/C \|\| NiFe-LDH@PTFE/NF | 0.107 | 0.011 | 0.090 |
| Pt/C \|\| NiFe-LDH/NF | 0.104 | 0.013 | 0.184 |
| Pt/C \|\| commercial IrO_2_/NF | 0.103 | 0.023 | 0.282 |

**References**

[1] G. Xiong, Y. Chen, Z. Zhou, F. Liu, X. Liu, L. Yang, Q. Liu, Y. Sang, H. Liu, X. Zhang, J. Jia, W. Zhou, *Advanced Functional Materials* **2021**, *31*, 2009580.

[2] Q. Wen, K. Yang, D. Huang, G. Cheng, X. Ai, Y. Liu, J. Fang, H. Li, L. Yu, T. Zhai, *Advanced Energy Materials* **2021**, *11*, 2102353.

[3] C. Li, Z. Zhang, R. Liu, *Small* **2020**, *16*, 2003777.

[4] H. Zhang, X. Li, A. Hähnel, V. Naumann, C. Lin, S. Azimi, S. L. Schweizer, A. W. Maijenburg, R. B. Wehrspohn, *Advanced Functional Materials* **2018**, *28*, 1706847.

[5] Y.-j. Wu, J. Yang, T.-x. Tu, W.-q. Li, P.-f. Zhang, Y. Zhou, J.-f. Li, J.-t. Li, S.-G. Sun, *Angewandte Chemie-International Edition* **2021**, *60*, 26829-26836.

[6] Y. Lin, H. Wang, C.-K. Peng, L. Bu, C.-L. Chiang, K. Tian, Y. Zhao, J. Zhao, Y.-G. Lin, J.-M. Lee, L. Gao, *Small* **2020**, *16*, 2002426.

[7] Z. He, J. Zhang, Z. Gong, H. Lei, D. Zhou, N. Zhang, W. Mai, S. Zhao, Y. Chen, *Nature Communications* **2022**, *13*, 2191.

[8] L. Dai, Z.-N. Chen, L. Li, P. Yin, Z. Liu, H. Zhang, *Adv. Mater.* **2020**, *32*, 1906915.

[9] Q. Wang, X. Huang, Z. L. Zhao, M. Wang, B. Xiang, J. Li, Z. Feng, H. Xu, M. Gu, *Journal of the American Chemical Society* **2020**, *142*, 7425-7433.

[10] Z. Pei, X. F. Lu, H. Zhang, Y. Li, D. Luan, X.-W. Lou, *Angewandte Chemie-International Edition* **2022**, *61*, e202207537.

[11] H. Yang, G. Vijaykumar, Z. Chen, J. N. Hausmann, I. Mondal, S. Ghosh, V. C. Nicolaus, K. Laun, I. Zebger, M. Driess, *Advanced Functional Materials* **2023**, 2303702.

[12] P. Liu, J. Wang, X. Wang, L. Liu, X. Yan, H. Wang, Q. Lu, F. Wang, Z. Ren, *International Journal of Hydrogen Energy* **2024**, *49*, 285-294.

[13] L. Li, P. C. Laan, X. Yan, X. Cao, M. J. Mekkering, K. Zhao, L. Ke, X. Jiang, X. Wu, L. Li, *Advanced Science* **2023**, *10*, 2206180.

[14] P. Fang, M. Zhu, J. Liu, Z. Zhu, J. Hu, X. Xu, *Advanced Energy Materials* **2023**, *13*, 2301222.

[15] T. Jiang, X. Jiang, J. Hnát, A. Michalcova, I. Biswas, R. Reissner, V. Kyriakou, F. Razmjooei, H. Liao, K. Bouzek, *Journal of Materials Chemistry A* **2022**, *10*, 23863-23873.

[16] Y. Zhao, Q. Wen, D. Huang, C. Jiao, Y. Liu, Y. Liu, J. Fang, M. Sun, L. Yu, *Advanced Energy Materials* **2023**, *13*, 2203595.

[17] H. Yang, P. V. Menezes, G. Dai, G. Vijaykumar, Z. Chen, M. Al-Shakran, T. Jacob, M. Driess, P. W. Menezes, *Applied Catalysis B: Environmental* **2023**, *324*, 122249.

[18] J. Chen, Y. Wang, G. Qian, T. Yu, Z. Wang, L. Luo, F. Shen, S. Yin, *Chemical Engineering Journal* **2021**, *421*, 129892.

[19] L. Wan, J. Liu, Z. Xu, Q. Xu, M. Pang, P. Wang, B. Wang, *Small* **2022**, *18*, 2200380.

[20] X. Wang, Z. Jiang, Y. Ma, X. Su, X. Zhao, A. Zhu, Q. Zhang, *Journal of Power Sources* **2024**, *591*, 233819.

[21] J. Abed, S. Ahmadi, L. Laverdure, A. Abdellah, C. P. O'Brien, K. Cole, P. Sobrinho, D. Sinton, D. Higgins, N. Mosey, *Adv. Mater.* **2021**, *33*, 2103812.

[22] L. Han, H. Yu, Z. Xiang, *Small* **2023**, *19*, 2304108.

[23] Y. S. Park, J. Yang, J. Lee, M. J. Jang, J. Jeong, W.-S. Choi, Y. Kim, Y. Yin, M. H. Seo, Z. Chen, S. M. Choi, *Applied Catalysis B: Environmental* **2020**, *278*, 119276.

[24] B. Chen, A. L. G. Biancolli, C. L. Radford, S. Holdcroft, *ACS Energy Letters* **2023**, *8*, 2661-2667.

[25] S. S. Jeon, J. Lim, P. W. Kang, J. W. Lee, G. Kang, H. Lee, *ACS Applied Materials & Interfaces* **2021**, *13*, 37179-37186.
